# Aggregation-Resistant, Turn-On-Off Fluorometric Sensing of Glutathione and Nickel (II) Using Vancomycin-Conjugated Gold Nanoparticles

**DOI:** 10.3390/bios14010049

**Published:** 2024-01-16

**Authors:** Atul Kumar Tiwari, Munesh Kumar Gupta, Hari Prakash Yadav, Roger J. Narayan, Prem C. Pandey

**Affiliations:** 1 Department of Chemistry, Indian Institute of Technology, Banaras Hindu University, Varanasi 221005, India; atulkumartiwari.rs.chy19@itbhu.ac.in (A.K.T.); hariprakashyadav.rs.chy19@itbhu.ac.in (H.P.Y.); 2 Department of Microbiology, Institute of Medical Sciences, Banaras Hindu University, Varanasi 221005, India; muneshg.micro@bhu.ac.in; 3 Joint Department of Biomedical Engineering, University of North Carolina, Chapel Hill, NC 27695, USA

**Keywords:** fluorescent gold nanoparticles, polyethyleneimine, antibiotic-conjugated gold nanoparticles, glutathione, fluorometric sensing of Ni^2+^

## Abstract

Glutathione (GSH) and nickel (II) cation have an indispensable role in various physiological processes, including preventing the oxidative damage of cells and acting as a cofactor for lipid metabolic enzymes. An imbalance in the physiological level of these species may cause serious health complications. Therefore, sensitive and selective fluorescent probes for the detection of GSH and nickel (II) are of great interest for clinical as well as environmental monitoring. Herein, vancomycin-conjugated gold nanoparticles (PEI-AuNP@Van) were prepared and employed for the detection of GSH and nickel (II) based on a turn-on-off mechanism. The as-synthesized PEI-AuNP@Van was ~7.5 nm in size; it exhibited a spherical shape with face-centered cubic lattice symmetry. As compared to vancomycin unconjugated gold nanoparticles, GSH led to the turn-on state of PEI-AuNP@Van, while Ni^2+^ acted as a fluorescence quencher (turn-off) without the aggregation of nanoparticles. These phenomena strongly justify the active role of vancomycin conjugation for the detection of GSH and Ni^2+^. The turn-on-off kinetics was linearly proportional over the concentration range between 0.05–0.8 µM and 0.05–6.4 μM. The detection limits were 205.9 and 90.5 nM for GSH and Ni^2+^, respectively; these results are excellent in comparison to previous reports. This study demonstrates the active role of vancomycin conjugation for sensing of GSH and Ni^2+^ along with PEI-AuNP@Van as a promising nanoprobe.

## 1. Introduction

Nickel (Ni) is a transition element that is widespread in low quantities in the environment; it is commonly found in soil, water, plant matter, and animal tissue. Besides serving as an indispensable trace element, it efficiently interacts with RNA and participates in the pigmentation process. The optimum concentration of Ni (II) in the human plasma ranges from 12 to 85 μgL^−1^. It is also used in industries such as oil hydrogenation, coal combustion, and catalysis nickel plating; in addition, it is used in diesel, tobacco, nickel-cadmium battery, cast iron, and heat-resistant steel production [1,2]. Pathophysiologically, excessive exposure to nickel in humans causes dermatitis, pneumonia, and lung cancer [3,4]. Over the past decade, industrial demand for nickel in a diverse range of metallurgical and catalytic processes has significantly increased. Simultaneously, the environmental and health concerns associated with nickel exposure have increased. Therefore, monitoring Ni (II) in environmental samples is critical to avoid nickel toxicity. Unlike Ni (II), glutathione (GSH) plays a role in human physiology and plant physiology [5]. Biothiols are a class of mercaptan with a sulfhydryl functional group; these materials are among the most significant antioxidants against cellular oxidative damage [6]. GSH is a tripeptide (glutamic acid–cysteine–glycine). It is a beneficial antioxidant thiol species in plant and mammalian cells that protects tissues and genetic material (e.g., DNA and RNA) from oxidative damage [7,8]. Imbalances and deficiencies of glutathione make cells prone to oxidative damage, resulting in a wide range of pathological situations, such as cancer, neurodegenerative disorders, and aging. To prevent GSH-induced physiological disorders, monitoring GSH in the human body is necessary.

Several instrumental techniques have been employed to quantify the Ni (II) in environmental samples, including spectrophotometry [9], atomic absorption spectrometry (AAS) [10], inductively coupled plasma-atomic emission spectrometry (ICP-AES) [11], as well as inductively coupled plasma-mass spectrometry (ICP-MS) [12]. It should be noted that these techniques are expensive, time-consuming, and require technical expertise for sample preparation before instrumental analysis. Similarly, the procedures for sensing and quantifying thiols in human samples vary. Human plasma [13], urine [14], and cell samples are required, followed by cell lysis [15]. Optical techniques are the most desirable among the various methods to detect and quantify thiols since they are easy to operate, sensitive, and efficient [16,17].

Metal nanoparticles, including gold, silver, and copper, have recently been explored in analytical chemistry as nanoprobes for detecting and quantifying a wide range of analytes [18,19]. Among these, the unique physicochemical properties (e.g., local surface plasmon resonance (LSPR)) of AuNPs and fluorometric sensing approaches have attracted researchers due to rapid probe synthesis, selective surface functionalization, rapid analysis, cost-effectiveness, and straightforward operation [20,21,22]. The surface functionalization approach utilizing gold nanoparticles (AuNPs) involves either receptor molecules or ligands that contain gold–nitrogen (Au-N) and gold–sulfur (Au-S) bonds [22]. Several research groups explored metal nanoparticles such as AgNPs [8], AuNCs [23], upconversion nanoparticles [24], and quantum dots [25] for the colorimetric detection of GSH. Park et al., 2013, developed a label-free and fluorescent turn-on sensor that was used for thiol (e.g., glutathione) detection [23]. This approach involved bovine serum albumin templated biogenic processing of gold nanoclusters and provided a limit of detection (LOD) of 9.4 nM. The assay involves blocking the Hg^2+^ induced fluorescence quenching activity of AuNCs via selective coordination of GSH with Hg^2+^ ions via trapping of Hg^2+^ by Hg–S interaction [23]. Ultimately, the fluorescence quenching activity of AuNCs is reversed via the selective coordination of Hg^2+^ ions to thiol.

Recently, our group [24,25,26] demonstrated the rapid and nano-geometry-controlled synthesis of silver, gold, and antibiotic-conjugated gold nanoparticles, exploiting the active role of PEI and formaldehyde/cyclohexanone. An advantage of PEI-stabilized synthesis of the noble metal nanoparticle is that amine groups may provide appropriate active sites for functionalization and facilitate surface changes toward the selective detection of various ions and biomolecules [27,28,29,30,31]. In the presented work, we described novel findings related to the turn-on-off sensing of GSH and Ni (II) using highly fluorescent, vancomycin-conjugated gold nanoparticles.

## 2. Materials and Methods

All of the reagents and materials were noted to be of analytical quality. Vancomycin (≥85%, CAS no. 1404-93-9), tetra-chloroauric acid trihydrate (HAuCl_4_.3H_2_O; 99.9%, CAS no. 16961-25-4), polyethyleneimine (50% *w*/*v* in H_2_O; Mw. 750k, CAS no. 9002-98-6), glutathione, BSA (Bovine serum albumin) and formaldehyde (36.0% in H_2_O) were obtained from Sigma Aldrich (Mumbai, India). Nickel sulfate, mercuric chloride (99.5%), potassium chloride (99%), nickel sulfate, magnesium chloride, sodium chloride (99%), ammonium chloride, tryptophan, tyrosine, and solvents were purchased from Merck Life Sciences (Bangalore, India). The other plasticware and glassware were acquired from Tarson (Mumbai, India).

### 2.1. Synthesis of Colloidal Vancomycin-Conjugated Gold Nanoparticles and Physical Characterization

The synthesis and surface tuning of PEI-stabilized gold nanoparticles were performed as described by Tiwari et al., 2023 [31]. Briefly, 200 μL (10 mM, working concentration) of HAuCl_4_.3H_2_O was mixed with an aqueous solution of vancomycin (40 μL, 2 mg/mL stock) in a 2 mL capacity glass vial, followed by stirring on a magnetic stirrer for 5–10 min. Further, an aqueous solution of PEI (polyethyleneimine) (30 μL of 4 mg/mL stock solution) was added, followed by continuous stirring and adding 30 μL of formaldehyde. This reaction mixture was stirred for the next 40–60 min at room temperature to obtain a dark red-pink color of PEI-AuNP@Van.

The physical characterization of the synthesized PEI-AuNP@Van included conventional as well as advanced instrumental techniques, including UV-VIS spectroscopic spectroscopy (Hitachi U- 2900 spectrophotometer; Tokyo, Japan) and transmission electron microscopy (FEI Tecnai G2 20 S Twin instrument; Hillsboro, OR, USA) measurements. A Malvern Nano Zeta Sizer (Malvern, UK) was used to acquire dynamic light scattering (DLS), zeta potential; an X-ray diffraction (XRD) spectrum was recorded using a Rigaku Mini-Flex 600 instrument (Tokyo, Japan). The particle size of gold nanoparticles was evaluated utilizing ImageJ software, version 1.54b 08 January 2023 (National Institutes of Health, Bethesda, MD, USA), followed by plotting a statistical graph on Origin 8.5 software (Northampton, MA, USA). Further, fluorescence emission (FL emission) spectra and a time-resolved photoluminescence spectrum were recorded on a Hitachi F7000 fluorescence spectrophotometer (Tokyo, Japan) and a WI-Tec alpha 300 RA instrument (Ulm, Germany), respectively. The CIE-1931 chromaticity diagram was calculated using an online server platform (https://sciapps.sci-sim.com/CIE1931.html, accessed on 14 October 2023).

### 2.2. Quantum Yield Determination of PEI-AuNP@Van

The fluorescence quantum yield of the PEI-AuNP@Van was determined as described previously [32]. Briefly, quinine sulfate was used as the fluorescent reference material at an excitation wavelength of 320 nm; it has a quantum yield of 0.54 [32]. We maintained the absorbance (below 0.1) and recorded the PL intensity of quinine sulfate (in 0.5 M H_2_SO_4_ at 320 nm excitation) and UV–visible absorbance. Analogous experiments were conducted with PEI-AuNP@Van under similar conditions, followed by the calculation of quantum yield by applying Equation (1); the obtained data are presented in Table 1.

(1) $$QY=QY_{ref}\frac{I}{A}\;\frac{A_{ref}}{I_{ref}}\;\frac{\eta^{2}}{\eta_{ref}^{2}}\;\;\;\;\;\;\;\;\;\;$$

where ‘*QY*’ represents the fluorescence quantum yield of the sample, ‘*I*’ denotes the integrated PL emission intensity at an excitation of 320 nm, ‘*A*’ denotes UV-Vis absorbance, ‘*η*’ denotes the refractive index of solvent (for water; 1.3), and ‘ref’ denotes the reference of the compound.

### 2.3. Experimental Setup for Fluorometric Sensing of Ni^2+^ and GSH

As described previously, fluorescence spectroscopic experiments were performed in ultra-purified HPLC grade water (Merck Life Sciences) [31]. First, 10 μL of synthesized vancomycin un-conjugated (PEI-AuNPs) and conjugated (PEI-AuNP@Van) nanoparticles were dispensed in a 1 cm quartz cuvette containing variable concentrations (from 0.05 to 51.2 μM) of aqueous solution of Ni^2+^ and GSH. The fluorescence spectra were recorded with a 5/10 slit width and with the application of a PTM voltage of 700, using an excitation wavelength of 320 nm. To validate the accuracy and consistency of the methodology, all of the measurements were repeated three times.

## 3. Results and Discussion

### 3.1. Synthesis and Characterization of Vancomycin-Conjugated Gold Nanoparticles

**Figure 1:**
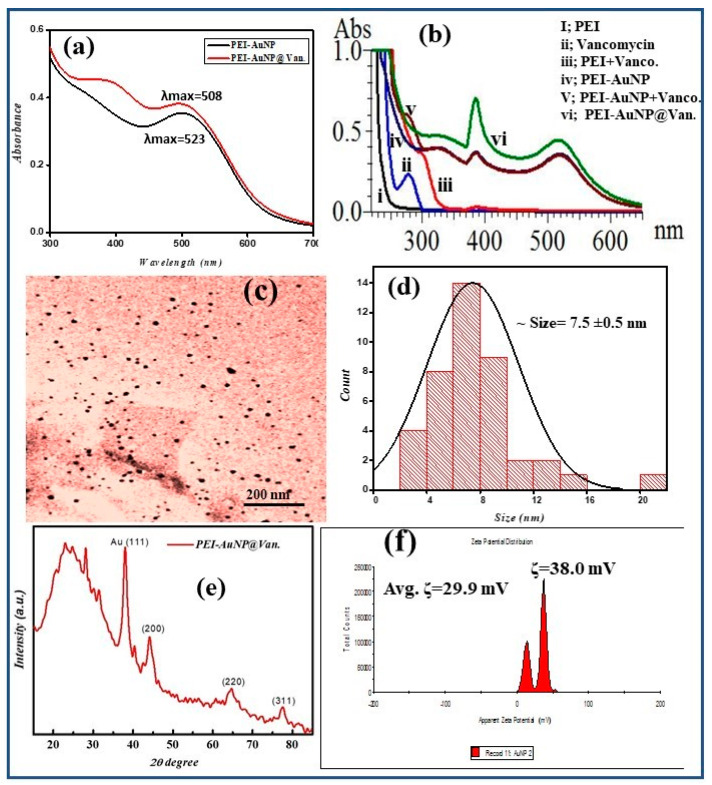
Representing the physical characterization of synthesized PEI-AuNP@Van. (**a**) UV–visible spectra along with un-conjugated gold nanoparticles (PEI-AuNPs), (**b**) corresponding UV-Vis spectra of all reactants and changes during the reaction, (**c**) TEM micrograph, (**d**) particle size distribution plot, (**e**) X-ray differactogram. Adopted from [31] © Tiwari et al., 2023; (**f**) zeta potential distribution.

**Figure 2:**
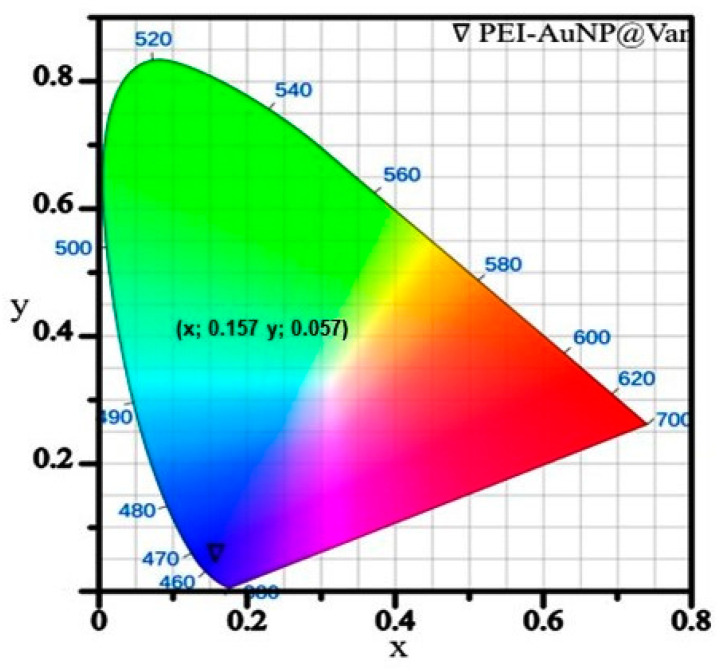
CIE-1931 coordinates of vancomycin-conjugated fluorescent AuNPs (PEI-AuNP@Van).

A single-step procedure optimized for the synthesis of vancomycin-conjugated gold nanoparticles was reported previously by Tiwari et al., 2023 [31]. The synthesized PEI-AuNP@Van nanoparticles showed absorption maxima at 508 nm (in comparison to unconjugated gold nanoparticles (PEI-AuNPs; 525 nm)) (as shown in Figure 1a). The interaction dynamics of vancomycin with other reactants was also established using UV-vis spectrometry (as demonstrated in Figure 1b). The origin of an intense peak at a wavelength of 384 nm in PEI-AuNP@Van represents the overlap of vancomycin COO- and PEI NH- groups [33]. No such peak was observed in unconjugated nanoparticles (PEI-AuNPs) (Figure 1b(v, vi), suggesting successful conjugation of vancomycin to the PEI molecules that further form a micelle to stabilize the gold nanoparticle and serve as a protection layer against agglomeration. The prepared PEI-AuNP@Van was 7.5 ± 0.5 nm in size (represented in Figure 1c,d). The lattice structure of the as-synthesized PEI-AuNP@Van was investigated using X-ray diffractometry between 10° to 80° 2θ degrees. Figure 1b shows peaks at 38.4°, 44.6°, 64.6°, and 77.7°, which correspond to the hkl values (111), (200), (220), and (311), respectively. It should be noted that the peak at 38.4° showed greater intensity than the other peaks, indicating that the Au atomic arrangement was in the face-centered cubic cell (FCC) structure (Figure 1e) [31]. As shown in Figure 1f, the recorded zeta potential of PEI-AuNP@Van demonstrated a bimodal distribution with ~29.8 mV, indicating a positive charge on the surface of PEI-AuNP@Van. Due to the greater magnitude of the zeta potential value, the dispersibility and fluorescence of the PEI-AuNP@Van in water were maintained for several months. Our findings demonstrate an excitation-dependent emission pattern over the 270–360 nm range in as-synthesized PEI-AuNP@Van, which was attributed to numerous trap surface states [31]. Upon excitation at 320 nm, PEI-AuNP@Van exhibited significant quantum yields of 15.40% compared to quinine sulfate (54% in 0.5 M H_2_SO_4_), which served as a standard. The quantum yield is attributed to the nano-geometry and functional activity of vancomycin. Considering its fluorescent properties, PEI-AuNP@Van has been identified as an appropriate nanoprobe for sensing chemicals and biological molecules. Fluorescence emission spectra and CIE chromaticity diagram coordinates (x 0.157, y 0.057) (Figure 2) indicate that PEI-AuNP@Van emits blue-color light when exposed to UV light (λ = 320 nm). Hence, vancomycin is naturally fluorescent; it provides an emission peak at ~350 nm when it is excited at 280–310 nm. However, after conjugation with gold nanoparticles, the emission spectrum shifted to 420 nm, demonstrating an alignment of emission with the characteristic fluorescence of bare gold nanoparticles, which enhances the fluorescence intensity of gold nanoparticles.

### 3.2. Effect of Salts, pH, and Time over Synthesized PEI-AuNP@Van

**Figure 3:**
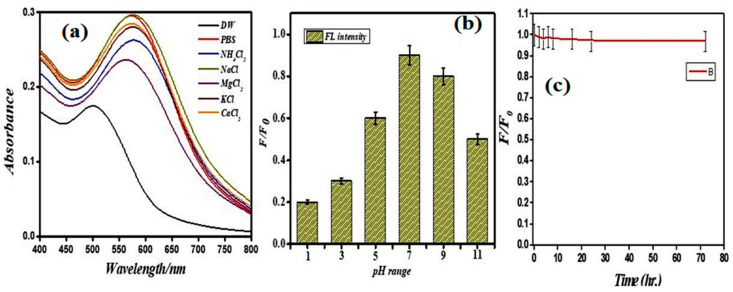
Stability of PEI-AuNP@Van in various salts (**a**) at variable pH (**b**) and the effect of time on fluorescence (**c**). Adopted from [31] © Tiwari et al., 2023.

To optimize the sensitivity and selectivity against Ni^2+^ and GSH, the stability of PEI-AuNP@Van on various parameters (including salt tolerance, pH, and the effect of time on the fluorescence property) was evaluated. Ion adsorption on the gold particle alters the gold surface plasmonic state due to alterations to the dielectric constant around the surface of the gold nanoparticle [34]. The specific cation adsorption from the salt solution on the gold nanoparticles alters their color from red to blue [34]. In the case of PEI-AuNP@Van, the effect of PBS, NH_4_Cl_2_, NaCl, MgCl_2_, KCl, and CaCl_2_ was evaluated and was in good agreement with previous reports, as demonstrated in Figure 3a. Due to the high zeta potential (cationic surface charge), the adsorption of anionic ions (chloride Ions) on the gold surface leads to agglomeration and shifting in the SPR absorption band. The absorption spectrum shifted from 508 to 580 nm under the influence of the tested salts, confirming that the agglomerated nanoparticles used to be ≤100 nm in size [35]. The fluorescence property of PEI-AuNP@Van was evaluated at various pH values (i.e., 1, 3, 5, 7, 9, and 11). The fluorescence intensity characteristics of the PEI-AuNP@Van were modulated as a function of pH; the intensity was the highest at a pH value of 6.5–7.0 (Figure 3b). Due to this finding, the neutral condition is more favorable; a pH of 7.0 was selected for the sensing experiments. Similarly, the fluorescence behavior of the probe was monitored over time (as demonstrated in Figure 3c). The fluorescence was maintained for 80 h without any significant loss.

### 3.3. Effect of GSH and Ni^2+^ on the SPR and Physical Properties of PEI-AuNP@Van

**Figure 4:**
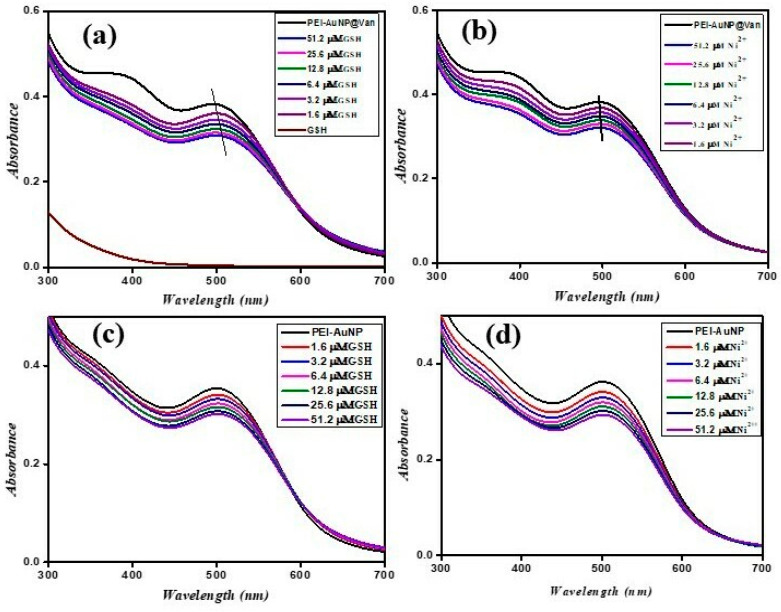
UV-Vis spectra of PEI-AuNP@Van and PEI-AuNPs upon adding different concentrations of GSH and Ni^2+^: (**a**) PEI-AuNP@Van in the presence of GSH; (**b**) PEI-AuNP@Van in the presence of Ni^2+^; (**c**) PEI-AuNPs in the presence of GSH; and (**d**) PEI-AuNPs in the presence of Ni^2+^.

**Figure 5:**
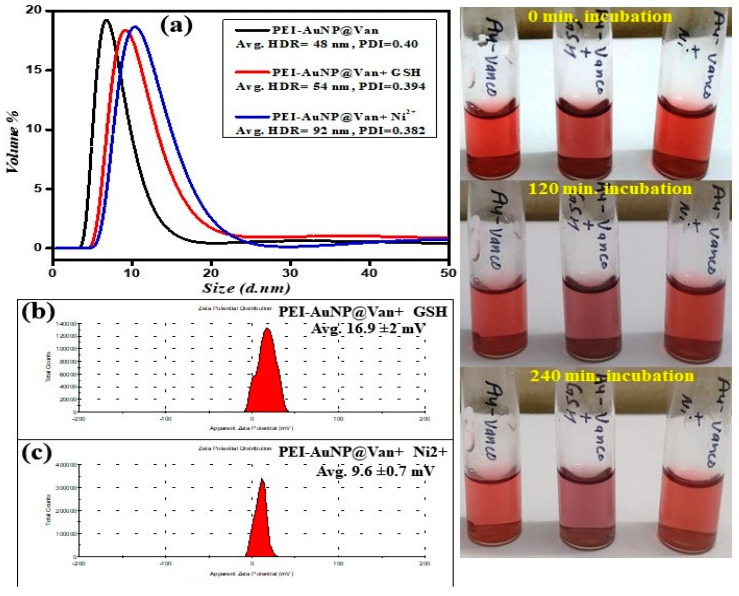
Changes in the fundamental physical properties of PEI-AuNP@Van after adding Ni^2+^ and GSH under similar conditions: (**a**) hydrodynamic radii, (**b**,**c**) zeta potential distribution.

The vancomycin-conjugated (PEI-AuNP@Van) and non-conjugated (PEI-AuNPs) displayed absorption peaks at 508 and 523 nm, respectively. However, PEI-AuNP@Van also shows an additional characteristic absorption peak at 384 nm (as represented in Figure 4a,b). The absorbance was recorded with different concentrations of 0.05 to 51.2 µM of GSH and Ni^2+^ under similar conditions against PEI-AuNPs and PEI-AuNP@Van (as demonstrated in Figure 4). With increasing concentrations of GSH and Ni^2+^, the PEI-AuNPs did not demonstrate any shift in the SPR band (Figure 4c,d). The increase of GSH concentration with PEI-AuNP@Van led to a systematic decrease in the absorbance at 384 and 508 nm along with a shifting in absorption peak at 508 to 515 nm (displayed in Figure 4a), which was in good agreement with previous reports [36]. In contrast to GSH, in Ni^2+^, less shifting was observed at 511 nm. The results led us to determine that some specific interaction of GSH and Ni^2+^ with vancomycin residues covered the gold nanoparticle core along with the polyethyleneimine moiety. Accordingly, the physical properties (DLS, zeta potential, and color) of PEI-AuNP@Van were validated after the addition of GSH and Ni^2+^, as indicated in Figure 5. A dynamic light scattering (DLS) analysis was performed on PEI-AuNP@Van, which had a mean diameter of 48 nm. After the addition of the highest concentration (51.2 µM) of GSH and Ni^2+^, the mean diameter was increased to 54 and 92 nm, respectively (as displayed in Figure 5a). Similarly, the recorded zeta potentials of PEI-AuNP@Van (Figure 1f) decreased to 16.9 and 9.6 mV, respectively (as shown in Figure 5b,c). Visual examination for 4 h of PEI-AuNP@Van after adding GSH showed a slight change in color from reddish to purple; however, no color change was noted in the case of Ni^2+^ under similar conditions (as represented in Figure 5d).

The above results indicate that the analytes, GSH and Ni^2+^, actively interacted with PEI-AuNP@Van; however, no interaction took place with PEI-AuNPs, confirming the dispensable role of vancomycin during interaction with analytes and as an anti-aggregation agent.

### 3.4. PEI-AuNP@Van mediated Fluorometric Sensing of GSH and Ni^2+^

**Figure 6:**
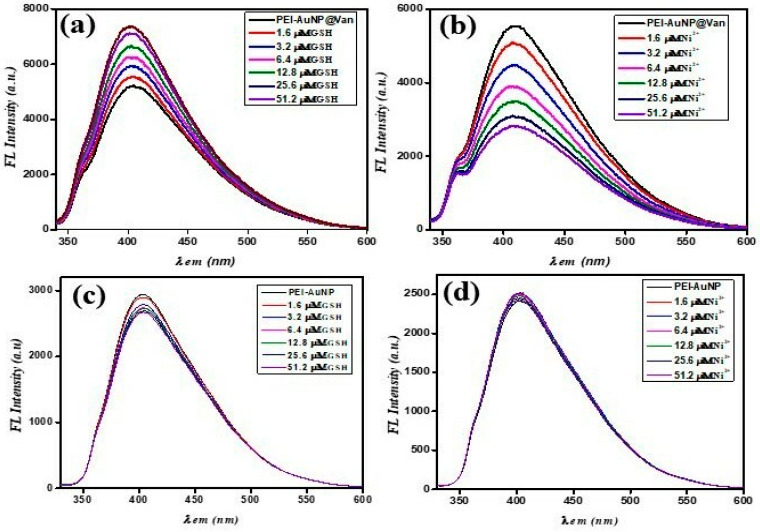
(**a**) PEI-AuNP@Van and PEI-AuNPs fluorescence emission spectra upon the addition of different concentrations (0.05 to 51.2 μM) of GSH and Ni^2+^. (**a**) PEI-AuNP@Van upon addition of GSH; (**b**) PEI-AuNP@Van upon addition of Ni^2+^; (**c**) PEI-AuNPs upon addition of GSH and (**d**) PEI-AuNPs upon addition of Ni^2+^ under similar conditions.

**Figure 7:**
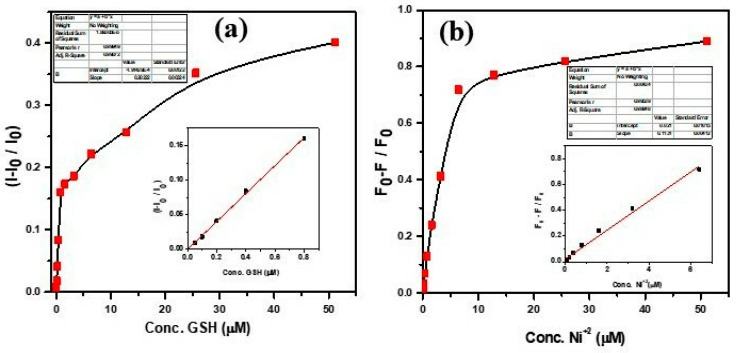
Stern–Volmer (S–V) and calibration curve of GSH (**a**) and Ni^2+^ (**b**). The insets represent the linearity curve and kinetic parameters.

To assess the sensitivity and selectivity of PEI-AuNP@Van as a fluorescence nanoprobe for detecting GSH and Ni^2+^, titration experiments were performed under ambient conditions at neutral pH. The fluorescence spectra of PEI-AuNP@Van (along with the unconjugated PEI-AuNPs system) were observed at an excitation wavelength of 320 nm at variable concentrations of GSH and Ni^2+^. As attributed in Figure 6a,b, the fluorescence intensity of PEI-AuNP@Van gradually increased (turn-on) upon the addition of GSH from 0.05 to 51.2 μM, while it was decreased (quenched/turn-off) in the presence of Ni^2+^ under similar conditions. Simultaneously, no significant increase or decrease was recorded in the unconjugated PEI-AuNPs system under similar conditions (Figure 6c,d). The kinetic parameters of PEI-AuNP@Van upon GSH-mediated turn-on and Ni^2+^-mediated turn-off were calculated by applying the modified Stern–Volmer equation as described previously [37,38]:

F_0_/F = 1 + KSV [GSH/Ni^2+^] (2)

where F is the FL intensity as a function of the concentrations of the enhancer/quencher [GSH/Ni^2+^], F_0_ is the FL emission intensity at [GSH/Ni^2+^] = 0, and KSV is the Stern–Volmer enhancement/quenching constant. The fluorescence intensity exhibits a well-linear relationship with the GSH concentration over a range of 0.05–0.8 μM (R^2^ = 0.9987) (Figure 7a inset); the linearity curve and kinetic parameters for Ni^2+^ concentration over a range of 0.05–6.4 µM (R^2^ = 0.9894) are provided in the Figure 7b inset. The limit of detection (LOD) was calculated (DL = 3.3xσ/S; in this equation, σ = slope and S = standard deviation) as 205.9 nM and 90.5 nM for GSH and Ni^2+,^ respectively. This finding was compared with previously reported nanomaterial-based fluorescence sensors (as shown in Table 2); PEI-AuNP@Van appears to be a promising probe for the detection of GSH and Ni^2+^.

### 3.5. Time-Resolved Fluorescence Lifetime Analysis

The fluorescence enhancement and quenching properties of PEI-AuNP@Van in the presence of GSH and Ni^2+^ were recorded using a double exponential function using this equation:

(3) $$D\;\left(t\right)=\sum_{i=1}^{n}ai\;exp(-\frac{t}{\tau i})$$

**Figure 8:**
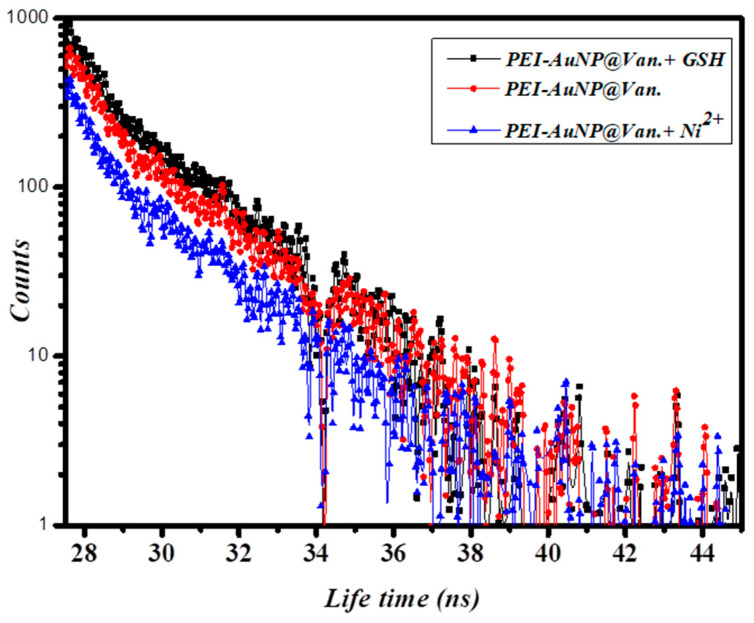
The fluorescence lifetime of PEI-AuNP@Van on incubation with and without Ni^2+^ and GSH.

Where *D* is fluorescence decay, (*ai*) associated pre-exponential factors, and *τi* is the fluorescence lifetimes of numerous fluorescent forms [31]. When no acceptor (GSH/Ni^2+^) was present, the PEI-AuNP@Van demonstrated an average lifetime of 3.20 ns (Figure 8). The average lifetimes were significant as 3.9 and 2.5 ns, respectively, after the addition of an electron donor and acceptor (i.e., GSH and Ni^2+^). The results indicate that when PEI-AuNP@Van transfers electrons to Ni^2+^, it acts as an electron acceptor. PEI-AuNP@Van accepted electrons from GSH (donor), which enhanced the fluorescence emission; vancomycin acted as a spacer molecule. Moreover, it was shown that the count was slightly reduced, demonstrating the least amount of agglomeration of PEI-AuNP@Van as confirmed by Figure 5a,d.

### 3.6. Selectivity of PEI-AuNP@Van for Ni^2+^ Ions and GSH

**Figure 9:**
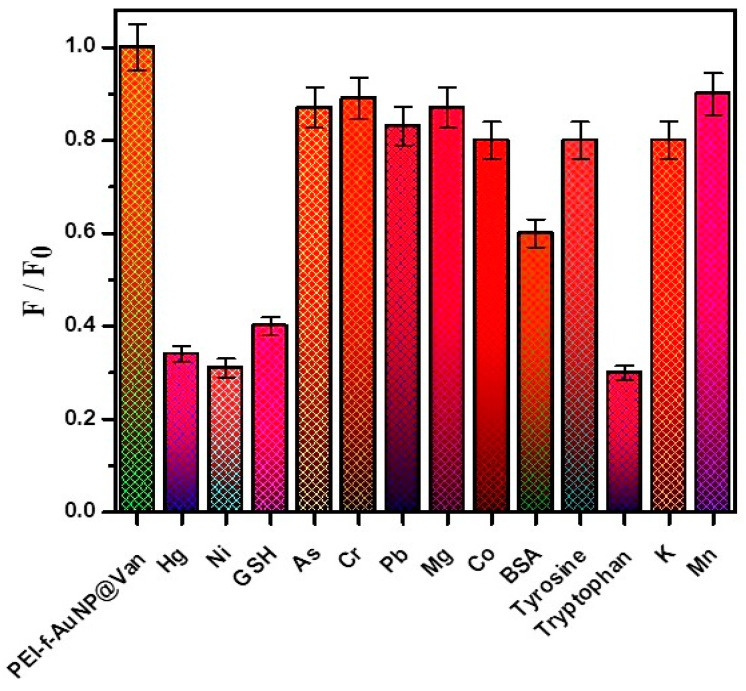
Selectivity of PEI-AuNP@Van nanoprobe towards heavy metal analytes and biomolecules.

A selectivity assay of PEI-AuNP@Van was evaluated towards 50 μM of Hg^2+^, Ni^2+^, Cr^3+^, Pb^2+^, As^3+^, Mg^2+^, Co^2+^, K^+^, and Mn^2+^ ions as well as biomolecules such as BSA, GSH, tryptophan, and tyrosine. As indicated in Figure 9, Hg^2+^, Ni^2+^, BSA, and tryptophan showed significant fluorescence quenching activity against the probe. The Ni^2+^ quenched the fluorescence of PEI-AuNP@Van around 75%; however, GSH enhanced the emission upto 50%. The other tested ions quenched only 10–20%.

## 4. Conclusions

This study examined polyethyleneimine stabilized nano-geometry controlled and rapid synthesis of vancomycin-conjugated highly fluorescent gold nanoparticles (PEI-AuNP@Van); sensing of GSH and Ni^2+^ with these materials under neutral pH was shown. The physical and chemical properties of PEI-AuNP@Van were evaluated using UV–vis spectroscopy, X-ray diffraction, transmission microscopy, photoluminescence spectroscopy, quantum yield determination, and chromaticity index. The size, crystallinity of PEI-AuNP@Van (~7.5 nm, spherical and FCC symmetry), and zeta potential value (~29.9 mV) suggest a nano-geometry controlled synthetic protocol with high stability under ambient conditions without loss of fluorescent characteristics. Further, the PEI-AuNP@Van nanoprobe demonstrated a strong selectivity and sensitivity for turn-on-off detection of GSH and Ni^2+^, respectively, with a detection limit of up to 205.9 and 90.5 nM. This study indicates possibilities for creating ultra-sensitive and low-cost PEI-AuNP@Van as a turn-on-off nanoprobe for the detection of GSH and Ni (II). Future, efforts to understand the functionality of PEI-AuNP@Van will involve assessing the nanoprobe with a variety of environmental (e.g., soil, water, and plant matter) and medical (e.g., animal and human tissue) samples.

## Figures and Tables

**Table 1:** The quantum yield of vancomycin-conjugated gold nanoparticles (at an excitation wavelength of 320 nm) with reference to quinine sulfate was calculated using Equation (1).

Sample	Integrated Intensity at 320 nm	Absorbance at 320 nm	Quantum Yield (%)
Quinine sulfate (reference)	65,840	0.04	54
PEI-AuNP@Van (sample)	23,465	0.05	15.40

**Table 2:** Comparison of various fluorescent and colorimetric sensors for GSH and Ni^2+^.

Nanoprobe	Method	Linear Range	Limit of Detection	References
Ce-MOF/AuNPs	Fluorescence (GSH)	0.2–32.5 μM	58 nM	[37]
QDs@SiO_2_@MnO_2_	FRET-based GSH sensing	0.16–0.48 mM	610 nM	[39]
AgNPs@SNCDs	Fluorescence (GSH)	8.35–200.5 μM	520 nM	[40]
CDs	Fluorescence (GSH)	1–150 μM	260 nM	[38]
MnO_2_ NS@Ru(bpy)3 ^2+^-UiO-66	Fluorescence (GSH)	0–300 μM	280 nM	[41]
Eu(DPA)_3_@Lap-Tris/Cu^2+^	Luminescent lanthanide-based GSH sensing	0.5–30 μM	162 nM	[42]
MMI-AuNPs	Colorimetric (GSH)	0.1–1.0 μM	12.0 nM	[43]
AuNPs@CV Nanosensors	SERS-based GSH sensing	0 − 24 μM	0.05 μM	[44]
PEI-AuNP@Van	Fluorescence (GSH)	0.05–0.8 μM	205.9 nM	Present work
Bio-AuNPs	Colorimetric (Ni^2+^)	0 to 1 mg/L	0.001 mg/L	[45]
Malonate AuNPs	Colorimetric (Ni^2+^)	10–500 ng mL^−1^	3 ng mL^−1^	[18]
Zwitterionic polypeptide capped gold nanoparticles (AuNPs-(EK)_3_	Colorimetric (Ni^2+^)	60–160 nM	34 nM	[46]
AuNPs-GSH	Colorimetric (Ni^2+^)	10–80 µM	10 µM	[47]
PEI-AuNP@Van	Fluorescence (Ni^2+^)	0.05–6.4 µM	90.5 nM	Present work

## Data Availability

The original contributions presented in the study are included in the article; further inquiries can be directed to the corresponding authors.

## Abbreviations

GSH	Glutathione
PEI-AuNPs	Un-conjugated polyethyleneimine stabilized gold nanoparticles
PEI-AuNP@Van	Vancomycin-conjugated polyethyleneimine-stabilized gold nanoparticles
DLS	Dynamic light scattering
